# Characteristics of associated craniofacial trauma in patients with head injuries: An experience with 100 cases

**DOI:** 10.4103/0974-2700.50742

**Published:** 2009

**Authors:** Prasad B Rajendra, Tony P Mathew, Amit Agrawal, Gagan Sabharawal

**Affiliations:** Department of Neurosurgery, K.S.Hegde Medical Academy, Deralakatte-575018, Mangalore, Karnataka, India

**Keywords:** Craniofacial trauma, facial fracture, head injury

## Abstract

**Background:**

Facial fractures and concomitant cranial injuries carry the significant potential for mortality and neurological morbidity mainly in young adults.

**Aims and Objectives:**

To analyze the characteristics of head injuries and associated facial injuries, the management options and outcome following cranio-facial trauma.

**Methods:**

This retrospective review was performed at Justice K. S. Hegde Charitable Hospital, and associated A. B. Shetty Memorial Institute of Dental sciences, Deralakatte, Mangalore. Following Ethical Committee approval, hospital charts and radiographs of 100 consecutive patients of cranio-facial trauma managed at the Department of Oral and Maxillofacial Surgery and Neurosurgery between January 2004 and December 2004 were reviewed.

**Results:**

Majority of the patients were in the 2nd to 4th decade (79%) with a male to female ratio of -8.09:1. Road traffic accidents were the common cause of craniofacial trauma in present study (54%) followed by fall from height (30%). Loss of consciousness was the most common clinical symptom (62%) followed by headache (33%). Zygoma was the most commonly fractured facial bone 48.2% (alone 21.2%, in combination 27.2%). Majority of patients had mild head injury and managed conservatively in present series. Causes of surgical intervention for intracranial lesions were compound depressed fracture, contusion and intracranial hematoma. Operative indications for facial fractures were displaced facial bone fractures. Major causes of mortality were associated systemic injuries.

**Conclusion:**

Adult males are the most common victims in craniofacial trauma, and road traffic accidents were responsible for the majority. Most of the patients sustained mild head injuries and were managed conservatively. Open reduction and internal fixation with miniplates was used for displaced facial bone fractures.

## INTRODUCTION

Actually transmit forces directly to neurocranium, resulting in more serious brain injuries.[1–4] In view of high incidence of closed head injury in facial fracture population, as well as the potential for mortality and neurological morbidity, the practicing oral maxillofacial surgeon should be cognizant of this condition including its management.[5] A clearer understanding of the patterns of facial injuries will also assist health care providers to plan and manage the treatment of traumatic facial injuries. Such epidemiological information can also be used to guide the future funding of public health programs geared toward prevention. In this study, we analyze epidemiology, clinical characteristics and management options in patients with cranio-facial trauma.

### Aims and objectives

The aim of present study were to analyze the characteristics of head injuries and associated facial injuries, to study the patterns of facial injuries in patients with head injury and to study the management options and outcome following cranio-facial trauma.

## MATERIALS AND METHODS

This retrospective review was performed at Justice K. S. Hegde Charitable Hospital, and associated A. B. Shetty Memorial Institute of Dental sciences, Deralakatte, Mangalore. Following Ethical Committee approval, hospital charts and radiographs of 100 consecutive patients managed for cranio-facial trauma at the Department of Oral and Maxillofacial Surgery and Department of Neurosurgery between January 2004 and December 2004 were reviewed. All clinical records, investigations and treatment charts were reviewed and data regarding age, gender, etiology and pattern of injuries, anatomic site and pattern of facial fractures, associated cranial injuries and treatment details and complications were analyzed. The craniofacial fractures were identified according to anatomic location, age, and sex of the patient, cause of injury, additional injuries and systemic injuries. The Ommaya classification of head injury was used, and patients with head injuries were categorized into three grades: mild, moderate, or severe categories (based on the period of loss of consciousness and amnesia).[6] Maxillofacial trauma included trauma of the craniofacial skeleton (extending from the frontal bone to the mandible). The types of cranial fracture were classified by anatomic location as frontal, sphenoid, temporal, parietal and occipital. Fractures of the facial skeleton[7] based on facial bone imaging were grouped as lower face (LF - mandible), mid face (MF - maxilla, nose, zygoma, and orbits) and upper face (UF - frontal). Systemic injuries were grouped into following categories as lacerations and abrasions, injuries to liver, kidney, bladder and bowels, hemothorax, pneumothorax, loss of vision, fractures, long bone fractures, fracture, and spinal cord injury. Patients with signs and symptoms of possible intracranial injury and/or facial bone fractures underwent computed tomography (CT). The final diagnosis of facial fracture was made by the attending radiologist. Brain trauma was handled by Neurosurgery Department, and complex facial fractures were repaired by the Oral and Maxillofacial Surgery Department.

## RESULTS

**Figure 1:**
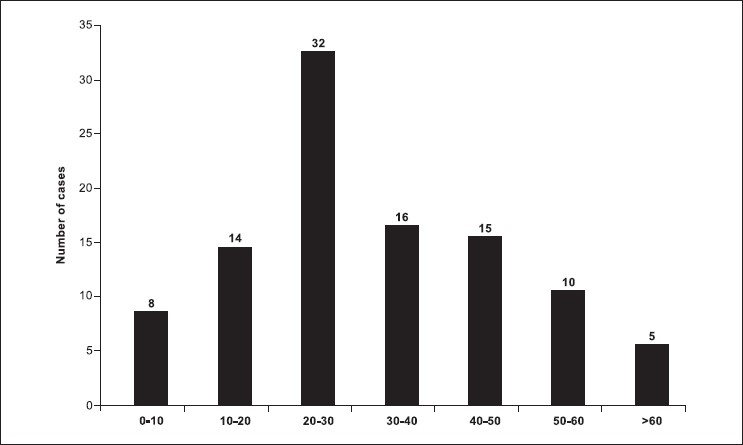
Age distribution

**Figure 2:**
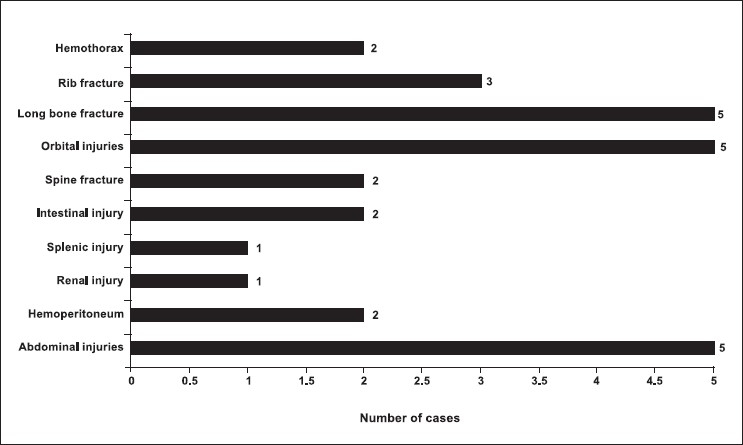
Distribution of associated injuries

**Figure 3:**
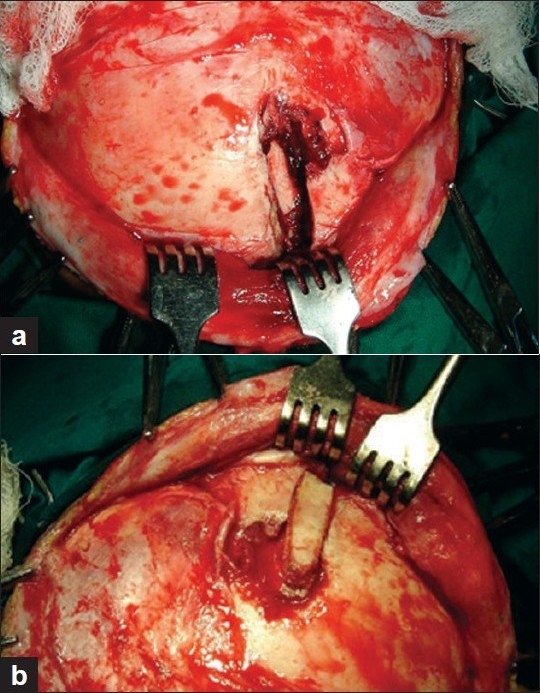
(a) Intra-operative image showing depressed fragment of frontal bone and associated fracture of orbital rim. (b) Intra-operative images: the depressed bone fragment was removed and the wound was cleaned; the bone fragment was replaced, and orbital margin was also re-constructed

**Figure 4:**
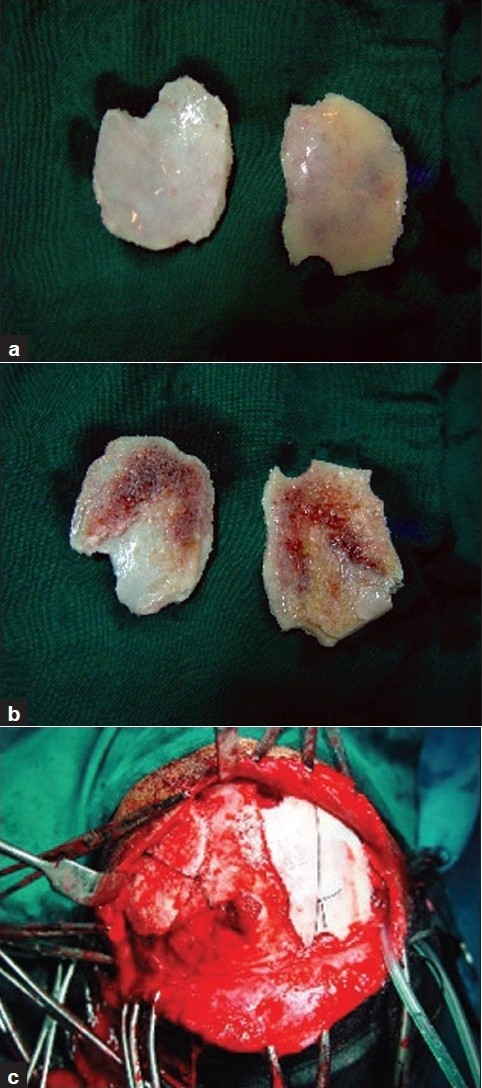
(a) Split calvarial graft harvested from frontal bone; outer aspect. (b) Split calvarial graft harvested from frontal bone; diploic aspect. (c) Intra-operative images of same patient (left) defect in the frontal bone, (right) split calvarial graft was used to repair the defect

**Figure 5:**
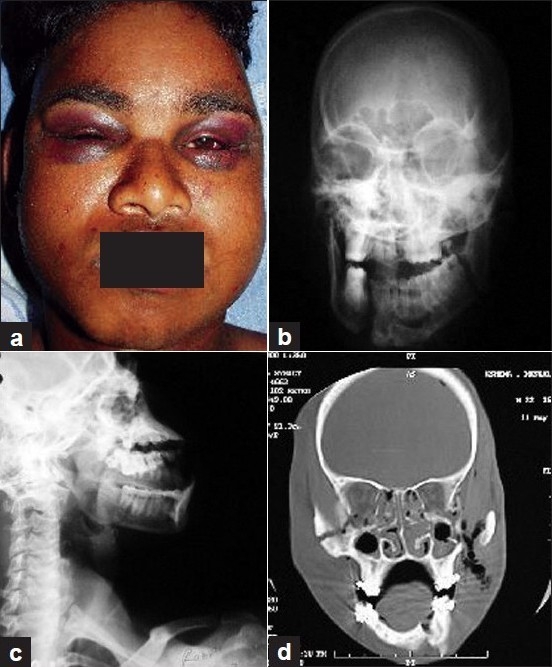
(a) Pre-operative images showing bilateral black eyes in a patient with craniofacial trauma. (b) X-ray skull: AP view of the same patient showing multiple fractures of facial bone including mandible. (c) X-ray skull: lateral view of the same patient showing multiple fractures of facial bone including mandible. (d) CT scan of the same patient showing details of facial bone fractures. Patient was treated surgically and internal fixation of bone fragments was performed

There were total 100 patients in present study who sustained cranial and/or maxillofacial injuries and majority of the patients were in 2nd to 4th decade (79%). Patients below 10 years (8%) and after 50 years (15%) were less commonly affected [Figure 1]. Eighty percent of patients in our study were male, and incidence of craniofacial injuries in female was 11 percent (male to female ratio - 8.09:1). Road traffic accidents were the commonest cause of craniofacial trauma in present study (54%) followed by fall from height (30%), assault (9%), occupational injuries (5%) and sports related injuries (1%) were less common causes of craniofacial trauma. One patient sustained injury in train accident. Loss of consciousness was the most common clinical symptom (62%) followed by headache (33%). Other clinical features were vomiting (27%), nasal bleed (30%) and oral bleed (10%). 33% patients had associated facial injuries, and the zygoma was the most commonly fractured facial bone (48.2%; alone 21.2%, in combination 27.2%). This was followed by mandible fracture (42%; alone 36%, in combination 6%). Maxilla was involved in 39.4% (alone 15.2%, in combination 24.2%). Loss of consciousness was commoner in patients with intracranial injury, and it was mainly due to concussion head injury (56%). In patients with facial fractures, association of loss of consciousness was most common with mandible fracture (14.7%) followed by zygomatic (8.2%) and maxillary (4.9%) fractures. Majority of the patients (93%) sustained mild head injury, two and five patients sustained moderate and severe head injury, respectively. All patients who sustained moderate (GCS 10-12, two cases) or severe head injury (GCS 6-9, five cases) had associated intracranial injury. All patient with facial bone fractures in present series sustained mild head injury(GCS 12-15). There was history of alcohol intake in 10 patients. Five patients sustained abdominal injuries (hemoperitoneum - 2, renal injury - 1, splenic injury - 1 and intestinal injury in 1 patient). Two patient sustained spinal injuries (fracture of D12 vertebrae and fracture of L1 vertebrae, respectively). Five patients sustained orbital injuries (orbital wall fracture in three cases). Other injuries were long bone fracture (5 cases), rib fracture (3 cases) and hemothorax (2 cases) [Figure 2]. Majority of patients had mild head injury and were treated conservatively in the present series. Causes of surgical intervention for intracranial lesions were compound depressed fracture of frontal bone (two cases - wound debridement and internal fixation) [Figures 3 a,b and 4 a-c], decompression of left temporal contusion (one case) evacuation of intracranial hematoma (extradural hematoma - 2 cases, chronic subdural hematoma - 4 cases) and suturing of scalp lacerations in 7 cases. Operative indications for facial fractures were displaced mandibular fractures (9 cases), zygomatic fractures (6 cases), maxilla fractures (3 cases) and zygomatic + maxilla fractures (7 cases) [Figure 5 a-d]. In all these patients, open reduction and internal fixation was performed, and fixation was achieved with miniplates. Isolated undisplaced fractures of mandible were treated conservatively with arch bar fixation alone.

### Complications and mortality

Two patients developed wound infection and were treated conservatively. Two patients expired in the present series. One patient had associated intra-abdominal injury (splenic rupture), and the other patient sustained pulmonary trauma and succumbed to hemothorax and aspiration.

## DISCUSSION

As in present study majority of the vast majority of head and concomitant facial injuries are experienced by young adult males ranging from 3:1 to as high as 11.1:1 in literature.[8–14] This high vulnerability of male gender for all types of trauma can be attributed to the facts that in our society male have more freedom to work outdoor and engage in risk-taking activities, making them more vulnerable to accidents and fall injuries. Road traffic accidents (RTA) are the commonest cause of craniofacial trauma in most of the series,[8,16–18] and this occur largely even in our circumstances also because of recklessness and negligence of the driver, poor maintenance of vehicles, often driving under the influence of alcohol or drugs and complete disregard of traffic laws.[19] Craniofacial trauma due to falls, altercations, sports, and warfare is less common in the literature.[14–20] Fall as one of the common cause of injury in our series can be attributed to the proximity of hospital to a coastal area where lot of peoples work and climb on the coconut trees. Interpersonal violence is becoming a major problem in many areas, and is attributed to the increasing use of alcohol and drugs.[17] In our study, we did not have many cases of such type of injuries; this low number of assault's victims may be explained by underreporting by patients.[8] Though we have not studied the causes of facial trauma in children, the majority of children sustain fall-related injuries mainly at home or playing places.[21,22] Other causes of facial injuries in children include automobile accidents[23] escalator-related entrapment injuries, particularly in younger children.[24] Recently, severe injuries of the facial skeleton and soft tissues similar to the adults have been described in children's.[25] Alcohol intoxication is a major confounding factor for reduced level of consciousness in patients with head injury, and CT scan is recommended when the level of alcohol intoxication is enough to reduce their GCS.[26] We performed the CT scan in all the cases where the history of alcohol intake was present. Isolated mandibular fractures are most common facial bone fractured (ranging from 12.9% to as high as 72.9%),[8–14–20,27,28] followed by midface (25.9% to 29.5%),[27,28] and among motorcyclists, maxilla, orbit, and nasal bones were the most frequently fractured bones.[29] The other frequently affected bones were the floor of the orbit and nasal bones.[30,31] However, in the present series, zygoma was the most commonly involved facial bone, followed by mandible and maxilla. Apart from maxillofacial fractures, high-velocity impacts may result in ruptures of intracranial vessels, leading to life-threatening intracranial hemorrhages.[32–35] Loss of consciousness can be manifestation of intracranial injury or concussion head injury;[17,36–38] however, loss of consciousness is less common with isolated facial fractures.[37] But at the same time, as in present series, all patients who sustain moderate or severe head injury also have associated intracranial injuries reflecting the severity and complexity of craniofacial trauma.[37] Although the incidence of spinal injuries was low in our series, in all the patients of craniofacial trauma, there should be a high index of suspicion of concomitant cervical spine fracture as up to 10% patients can have associated spinal injuries.[1,2] Although they represent serious injuries, the workup and treatment of facial fractures is often delayed until more life-threatening problems have been addressed, such as the establishment of an adequate airway, hemodynamic stabilization, and the evaluation and treatment of other more serious injuries of the head, chest and skeleton.[39] Present series signifies that majority of the patients sustain mild head injury and could be managed conservatively.[40,41] Indications for surgical intervention include compound depressed skull fractures, traumatic intracranial hematomas, contusion and suturing of scalp lacerations.[41] Our management approaches to facial fractures were according to the guidelines described in the literature, including repositioning of the displaced fracture segments into anatomic position, with a focus on the lattice supports in relation to each other and to the cranial base.[42–47] Undisplaced fractures were managed conservatively, and in displaced fractures, open reduction and internal fixation with miniplates was used. The rigid stabilization of the vertical and horizontal facial fracture helped to support and withstands the forces of mastication,[43,48–52] and conservative methods for undisplaced fractures provided acceptable functional and aesthetic results.[20,28,52,53] The incidence of complications in present series was low, and in literature, incidence of post-surgical complications ranges from 5%-5.7%, including infection, asymmetry, and malocclusion.[20,28,54] Mortality rate in our series is 2%, and the major cause of this mortality is associated systemic injuries and pulmonary infection.[54]

### Limitations

The present study works on the assumption that the given history was an accurate representation of the events. However, because of the retrospective nature of the study, it has inherent limitations that could be due to gaps in information and incomplete records, the accuracy of the original examination and documentation.

## CONCLUSIONS

The management of fractures to the face remains a challenge for oral and maxillofacial surgeons, demanding both skill and a high level of expertise. In summary, young adult males were the commonest victims of road traffic accidents and usually sustain mild head injuries. Management of the more serious intracranial and life-threatening injuries takes the priority; open reduction and internal fixation with miniplates is recommended for displaced facial bone fractures. A well planned prospective study will provide a clearer understanding of the patterns of facial injuries in patient with head injuries, and this epidemiological as well as clinical information will assist health care providers to plan the management of such injuries and will also guide the future funding of public health programs, particularly to develop prevention strategies.
